# A Feature Extraction Method for Seizure Detection Based on Multi-Site Synchronous Changes and Edge Detection Algorithm

**DOI:** 10.3390/brainsci13010052

**Published:** 2022-12-27

**Authors:** Xiang Gao, Yufang Yang, Fang Zhang, Fan Zhou, Junming Zhu, Jie Sun, Kedi Xu, Yaowu Chen

**Affiliations:** 1 Institute of Advanced Digital Technology and Instrument, Zhejiang University, Hangzhou 310027, China; 2 Binjiang Institude of Zhejiang University, Hangzhou 310027, China; 3 Qiushi Academy for Advanced Studies (QAAS) and Department of Biomedical Engineering, Zhejiang University, Hangzhou 310027, China; 4 Zhejiang University Embedded System Engineering Research Center, Ministry of Education of China, Zhejiang University, Hangzhou 310027, China; 5 Department of Neurosurgery, Second Affiliated Hospital of Zhejiang University, Hangzhou 310027, China; 6 Department of Neurosurgery, Ningbo First Hospital, Ningbo 315010, China; 7 Zhejiang Provincial Key Laboratory of Cardio-Cerebral Vascular Detection Technology and Medicinal Effectiveness Appraisal, Key Laboratory of Biomedical Engineering of Education Ministry, Zhejiang University, Hangzhou 310027, China; 8 MOE Frontier Science Center for Brain Science and Brain-Machine Integration, Zhejiang University, Hangzhou 310027, China; 9 Zhejiang Provincial Key Laboratory for Network Multimedia Technologies, Zhejiang University, Hangzhou 310027, China

**Keywords:** seizure detection, feature extraction, magnitude-squared coherence map, Canny edge detection algorithm, intracranial EEG

## Abstract

Automatic detection of epileptic seizures is important in epilepsy control and treatment, and specific feature extraction assists in accurate detection. We developed a feature extraction method for seizure detection based on multi-site synchronous changes and an edge detection algorithm. We investigated five chronic temporal lobe epilepsy rats with 8- and 12-channel detection sites in the hippocampus and limbic system. Multi-site synchronous changes were selected as a specific feature and implemented as a seizure detection method. For preprocessing, we used magnitude-squared coherence maps and Canny edge detection algorithm to find the frequency band with the most significant change in synchronization and the important channel pairs. In detection, we used the maximal cross-correlation coefficient as an indicator of synchronization and the correlation coefficient curves’ average value and standard deviation as two detection features. The method achieved high performance, with an average 96.60% detection rate, 2.63/h false alarm rate, and 1.25 s detection delay. The experimental results show that synchronization is an appropriate feature for seizure detection. The magnitude-squared coherence map can assist in selecting a specific frequency band and channel pairs to enhance the detection result. We found that individuals have a specific frequency band that reflects the most significant synchronization changes, and our method can individually adjust parameters and has good detection performance.

## 1. Introduction

Epilepsy seizures are brain disorders predominantly characterized by recurrent and unpredictable interruptions in normal brain function [1], which affect over 70 million people worldwide, with an incidence of 2.4 million per year [2,3,4]. Seizure detection is an important method to study the physiological mechanism, development process and treatment of epilepsy and has been widely used in clinical practice and scientific research. However, the most common detection method depends on the experience of clinical doctors and technicians, which places a burden on personnel and resources, and detection efficiency is subjective. Therefore, many automatic detection methods have been developed to help doctors save time and improve efficiency. For example, seizure signals can be automatically detected by finding the changes in the amplitude of the Electroencephalogram (EEG) signal. But there are still some problems with automatic seizure detection methods: the performance of detection methods in terms of accuracy, false alarm rate, and time delay needs to be improved; the computational complexity of some algorithms is high and difficult to be truly implemented; the detection features sometimes are not well adapted to different epilepsy seizure types. Therefore, further research is needed to improve the effectiveness of epilepsy detection.

In seizure detection algorithms, proper signal feature selection is the key factor in improving the efficiency of the detection result. Many features of single-channel seizure detection methods have been widely used, such as line length [5], amplitude [6], spectral features [7], time-frequency domain features [8,9,10], and features relating to information theory, such as approximate entropy [11]. Some features can achieve excellent performance but consume many computing resources, such as machine learning methods [12,13]. Some are preferable for online detection, with high computational efficiency [14], but the detection effect is not satisfactory, such as in linear detection methods. Synchronization is also a common feature in a multi-site method of seizure detection. The most used method for synchronization calculation is phase synchronization (PC) [15], which measures the interactions between rhythmic signals by detecting the instantaneous phase, and is unaffected by signal amplitude [16]. Depending on the position of the implanted electrode, the calculated synchronization results may increase [16,17,18] or decrease [19,20]. Further, synchronization changes between channels in different frequency bands have been found in recent studies [18,21,22]. In the PC method, it is difficult to observe synchronization changes in the full frequency band.

Another method to observe the synchronization change between signals in the frequency domain is called magnitude-squared coherence (MSC): a normalized cross-spectral density function that measures the strength of association and relative linearity between two stationary stochastic processes on a scale from zero to one [23]. In frequency-domain objective response detection, researchers compared MSC to PC using simulations with specific signal-to-noise ratios (SNRs) and a varying number of subaverages, and MSCs were superior to PC [24]. The MSC method is widely used in the fields of signal detection, time delay estimation, and SNR estimation [25,26,27,28], such as in human auditory models [29], electrocardiograms (ECGs), EEGs, magnetoencephalography (MEG) analysis [30,31,32] and seismic waves [33]. However, the current utilization of MSCs is limited in the frequency domain. MSCs only perform the calculation on a selected frequency, making it difficult to obtain a full view of the signals.

In this study, based on the existing MSC methods, we extended the MSC method to the time-frequency domain by drawing an MSC map to detect the synchronization change of two local field potential (LFP) signal channels. We used Canny edge detection algorithm to obtain information regarding the frequency band and when synchronization increases. We subsequently selected the frequency band where the synchronization change was the most apparent and calculated the maximal cross-correlation coefficient curve of the selected channel pairs after filtering. We used the average value (AVG) of correlation coefficient curves as the feature to detect seizure onset and added standard deviation (STD) as a parameter to reduce false alarms. This study focused on synchronization changes in the limbic system in a chronic spontaneous temporal lobe epilepsy (TLE) animal model. We found that individuals have a specific frequency band that reflects the most significant synchronization changes. Compared with other seizure detection features, the results showed that our features can individually adjust parameters and has good detection performance.

## 2. Materials and Methods

### 2.1. Animal Model and Data Acquisition

All surgical and experimental procedures were approved by the Animal Care and Use Committee of Zhejiang University. Pilocarpine-induced chronic TLE rat model was adopted in this study since this model closely resembles TLE in human. The procedure of inducing a chronic spontaneous TLE model is similar to our previous work [34]. Six adult Sprague-Dawley rats weighing about 250 g were injected with lithium chloride (12.7 mg/100 g i.p.) intraperitoneally. After 24 h, rats were treated with pilocarpine (3.5 mg/100 g i.p.) 30 min after being treated with atropine sulfate and supplemented by an extra half dose of pilocarpine after 30 min if status epilepsy (SE) was not observed. Once rats had SE status lasting over 90 min, a dose of diazepam (2 mg/100 g i.p.) was given to the rats to terminate the SE.

One week after drug induction, the rats in which spontaneous seizures were observed were selected for electrode implantation. All the rats were implanted with electrodes in the bilateral subiculum (SUB, AP: −6.0, ML: 3.0, DV:−3.0), CA1(AP: −3.6, ML: 2.0, DV: −3.0), and CA3 (AP: −4.2, ML: 3.0, DV: −3.7) of the hippocampus and anterior nucleus of the thalamus (ANT, AP: −1.5, ML: 1.5, DV: −5.6), and three rats had four more electrodes implanted, in the bilateral dentate gyrus (DG, AP: −5.0, ML: 3.2, DV: −3.6) and amygdala (AMG, AP: −2.4, ML: 4.8, DV: −8.8). The rats recovered for a week after surgery before being connected for long-term signal recording. One rat died two weeks after recording. The remaining five rats were included in the experiments.

A homemade recording system was used according to a previous study [4]. This system contains a front-end recording (RHD2132 Intan Tech, LLC, Los Angeles, CA, USA) to transform neural electrical signals into digital signals. A field-programmable gate array (FPGA) module (XEM6010-LX45, Opal Kelly, Inc., Portland, OR, USA) was used for signal processing and system control. Each channel’s LFP signal was recorded with a sample rate of 1 kHz and 16-bit resolution. The FPGA implemented a second-order IIR band stop filter of 50 Hz to filter out the power-supply noise. The data were then sent to an onboard secure digital card to be saved for subsequent analyses.

### 2.2. Construction of MSC Maps

**Figure 1:**
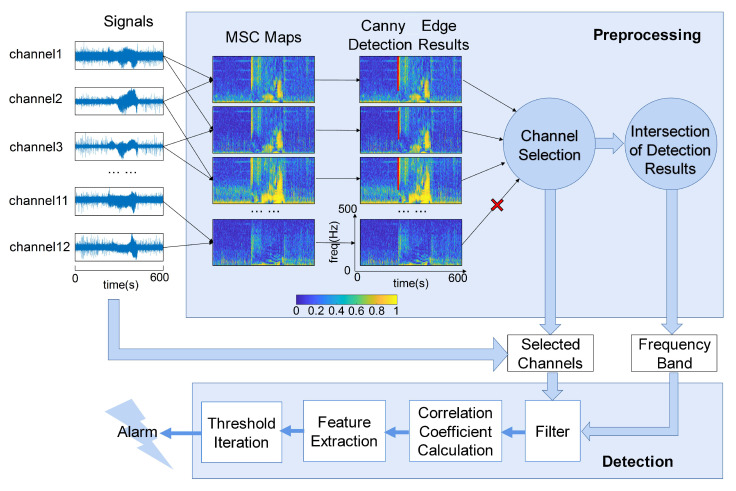
The process of multi-site seizure detection method. The processing is divided into two steps: preprocessing and detection. In preprocessing step, 12-channel signal fragments are first calculated between every two channels to get MSC maps. The edge detection is then performed by the Canny’s edge detection algorithm, and the result is shown as the red line in the figure. Select the original signals of the channel pair with detected edges for subsequent detection. Select the intersection of detection results as the frequency band. In detection step, the selected channels are filtered with the selected frequency band, and the seizure detection is completed through the steps of correlation coefficient calculation, feature extraction and threshold iteration.

As shown in Figure 1, the signals were calculated using the preprocessing and detection steps for seizure detection.

In the preprocessing step, the MSC map between every two channels was initially calculated to obtain the change in synchronization of different brain areas. In contrast to the phase coherence method, MSC is a power spectral density function that contains both amplitude and frequency information. Therefore, the MSC map of brain signals may reflect more seizure information [35]. The calculation formula for the MSC is as follows:

(1) $$C_{xy}\left(f\right)=\frac{{\left|P_{xy}\left(f\right)\right|}^{2}}{P_{xx}\left(f\right)P_{yy}\left(f\right)}$$

where $P_{xx}\left(f\right)$ and $P_{yy}\left(f\right)$ are the power spectral densities of signals *x* and *y*, $P_{xy}\left(f\right)$ is the cross-spectral density of signals *x* and *y*. The $C_{xy}\left(f\right)$ indicates the coherence between *x* and *y* at each frequency. The result is between 0 and 1. Our study estimated the MSC function using Welch’s overlapped average periodogram [36]. The Hamming window was used to divide the signal into segments with a 50% overlap on each segment.

To construct a time-frequency scale MSC map to illustrate the change in coherence on the time scale, we split the signals by a 400 ms window (overlap 200 ms, minimum resolvable frequency fmin=2.5 Hz) and analyzed MSC results for every segment. Then, we spliced each result in the direction of the time series and obtained an MSC map with time on the horizontal axis and frequency on the vertical axis, as shown in Figure 1.

### 2.3. Canny Edge Detection Algorithm

The Canny edge detection algorithm was applied to determine the MSC map’s earliest and strongest coherence change area. Canny edge detection is a method of smoothing and derivative, and optimization approximation is obtained based on the measurement of SNR and location prodseuct [37]. The basic steps for the Canny edge detection algorithm are as follows [38,39]:

(1) The original image is smoothed. The Gaussian filter is usually used, and in our study, the σ of the Gaussian filter was set to $\sqrt{2}$. The 2-D Gaussian filter function is as follows:

(2) $$h\left(x,y,\sigma \right)=\frac{1}{2\pi \sigma^{2}}e^{-\frac{x^{2}+y^{2}}{2\sigma^{2}}}$$

(2) Calculate the magnitude and direction of the gradient using the finite-difference of the first-order partial derivative. The x direction first-order partial derivative:

(3) $$G_{x}=\frac{\left[f(x+1,y)-f(x,y)+f(x+1,y+1)-f(x,y+1)\right]}{2}$$

The *y* direction first-order partial derivative:

(4) $$G_{y}=\frac{\left[f(x,y+1)-f(x,y)+f(x+1,y+1)-f(x+1,y)\right]}{2}$$

The magnitude of the gradient:

(5) $$M\left[x,y\right]=\sqrt{G_{x}{\left(x,y\right)}^{2}+G_{y}{\left(x,y\right)}^{2}}$$

The direction of the gradient:

(6) $$\theta \left[x,y\right]=\arctan\left[G_{x}\left(x,y\right)/G_{y}\left(x,y\right)\right]$$

(3) Non-maximum suppression: each pixel, M[x,y], is compared with its two neighboring pixels in the direction of the gradient θ[x,y]. If pixel M[x,y] is smaller by comparison, then M[x,y]=0.

(4) A dual-threshold algorithm is used to detect and connect the edges. We set the high threshold value to 0.90 to remove most of the noise. The low threshold value is set to 0.4 times the high threshold value to add a connection between the edges.

As shown in Figure 1, the most dramatic correlation changes (red line) in the MSC map were detected, demonstrating the frequency range with the strongest correlation between the two channels. Other three different edge detection algorithms were also compared in this study, including Roberts, Sobel, and Prewitt. The comparison results are shown in Appendix A.

Under a uniform threshold, not all edges can be detected by the Canny edge detection algorithm in MSC maps, such as channels 11 and 12 in Figure 1. It indicated that some channel pairs do not increase visibly in correlation with the other. To screen out proper channels for detection, we selected a 5-s segment in each MSC map that contained the seizure onsets. We quantified the results by summing all pixel values detected by the Canny edge detection algorithm. We ranked the quantized results from largest to smallest and selected the first ten channel pairs as the frequency band and seizure detection channels. Physically, the edges were the frequency bands of the increment of the correlation. We intersected the edge of the selected channel pairs to find the common frequency band for later detection.

### 2.4. Calculation of Maximal Cross-Correlation Coefficient

The calculations of MSC and Canny edge detection algorithm were complicated, and detection cannot be performed promptly. Therefore, the maximal cross-correlation coefficient was further applied in the detection step as a synchronization indicator to reduce computing resources and improve detection efficiency.

The selected signals were passed with zero-phase digital filters, and the frequency range was decided upon in the preprocessing step. As in the construction of MSC map, we split the signals with a 400 ms window and an overlap of 200 ms. We subsequently calculated the maximal cross-correlation coefficient in each window and arranged the results in the time series to construct the cross-correlation curves. The maximal cross-correlation coefficient was calculated for every channel pair as follows:

(7) $${\hat{R}}_{xy}\left(n\right)=\frac{1}{N}\sum_{m=0}^{N}x^{*}\left(m\right)y\left(m+n\right)$$

where x(m) and y(m) are the discrete signal sequences, *m* is the number of each function value appearing in the sequence, and *n* is the time lags between x(m) and y(m). *N* is the length of signal sequence. ${\hat{R}}_{xy}\left(n\right)$ can be normalized between 0 and 1 using the following formula:

(8) $${\hat{R}}_{xy,\;\mathrm{normalized}\;}\left(n\right)=\frac{{\hat{R}}_{xy}\left(n\right)}{\sqrt{{\hat{R}}_{xx}\left(0\right){\hat{R}}_{yy}\left(0\right)}}$$

${\hat{R}}_{xy,\;\mathrm{normalized}\;}\left(n\right)$ is the sequence associated with the time lags, where the maximal value is the maximal cross-correlation coefficient.

According to the Wiener–Khinchin theorem, the cross-correlation calculation results in the time domain can be transformed into the product form in the frequency domain:

(9) $${\hat{P}}_{xy}\left(f\right)=\sum_{-\infty }^{+\infty }{\hat{R}}_{xy}\left(n\right)e^{-i2\pi fn}=X^{*}\left(f\right)Y\left(f\right)$$

${\hat{P}}_{xy}\left(f\right)$ is the result of cross-spectral density of signal *x* and *y*. Therefore, the cross-correlation results can represent the MSC results.

After calculating the maximal cross-correlation coefficient curves of selected channel pairs, we extracted the AVG of these maximal cross-correlation coefficient curves as a detection feature.The AVG value represents the overall enhancement of correlation, but is susceptible to common-mode noise. Therefore, it is necessary to add ten channel pairs with insignificant correlation enhancement, and calculate STD with the previously selected channel pairs. It is an important parameter to reduce false alarms.

### 2.5. Threshold Iteration

**Figure 2:**
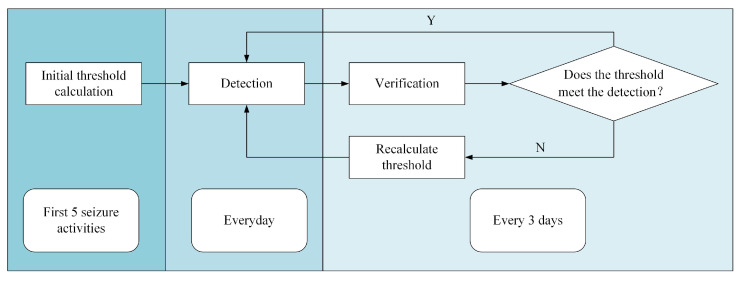
Flow chart of the threshold iteration process.

This study aimed to find a stable way to identify the starting time point of seizure activity. Two parameters, AVG and STD, were introduced to characterize the threshold of the seizure start time point. We used the initial five seizure activities of each rat to calculate the initial threshold using the following formula:

(10) Th=u+3∗sd

where *u* is the average value of the AVG and STD curves, and sd is the standard deviation of the AVG and STD curves. We verified the reliability of the thresholds every three days, and the data from every third day was used for the iterative calculation of thresholds. If the initial threshold detected all seizure activities, and the false alarm rate was less than ten times per hour, the thresholds were reserved for later detection. If one or more seizure activities were not detected, or if the false alarm rate was more than ten times per hour, the threshold was recalculated based on the current AVG and STD data. The updated thresholds were subsequently used to detect seizures within the last data and ensure that all seizure activities could be detected. If the thresholds were valid, they were reserved for later detection (Figure 2).

### 2.6. Comparison

One of the multi-channel features, mean phase coherence (MPC) [15] was used to compare the effect with our method using the same data sets. MPC is a frequency-domain analysis method to quantify the degree of phase synchronization for two time series, *x* and *y*, defined as [20]:

(11) $$R\left(x,y\right)=\left|\frac{1}{N}\sum_{j=0}^{N-1}e^{i\left[\phi_{x}\left(j\Delta t\right)-\phi_{y}\left(j\Delta t\right)\right]}\right|$$

where 1/Δt is the sample rate of the discrete time series of length *N*. *R* is restricted to the interval [0, 1]. (When two signals are synchronized, *R* reaches the value of 1 and inversely reaches the value 0.) The $\phi_{a}$ and $\phi_{b}$ are defined as the instantaneous phases for signals *x* and *y*:

(12) $$\phi_{x}=\arctan\frac{\tilde{x}}{x}$$

where $\tilde{x}$ is the Hilbert transform of the signal *x*.

In addition, the maximal cross-correlation coefficient (CC) feature [40,41] without frequency band selection and channel selection is also added to the comparison. We also selected 7 common single channel features for comparison, including amplitude (AMP), line length (LL), variance (VAR), maximum slope (MSP), total power (TP), power spectral ratio (PSR) and approximate entropy (ApEn). The detection thresholds in the nine comparison features were treated the same way as ours. The calculation formulas refer to Appendix B.

### 2.7. Data Analysis

The data and statistical analyses were performed using MATLAB R2020b software (The Mathworks, Natick, MA, USA). Continuous variables with normal distribution are presented as mean ± standard deviation; non-normal variables are reported as medians. All recorded seizure activities were reviewed and labeled by experienced neurologists. The seizure detection efficiency is evaluated by detection rate, detection delay, and false alarm rate. Detection rate is calculated as the rate of automatic detection number and total seizure number. Detection delay is calculated as the difference between automatically detected time and manually marked time. False alarm rate is calculated as the number of false positives per hour.

## 3. Results

### 3.1. Statistics of Seizure Activities

The number of intercepted seizure segments of the five rats was shown in Table 1. Experienced neurologists reviewed seizure activities from recorded brain signals combined with videos reviewed. A total of 206 seizure segments were identified.

### 3.2. Analysis of MSC Maps

**Figure 3:**
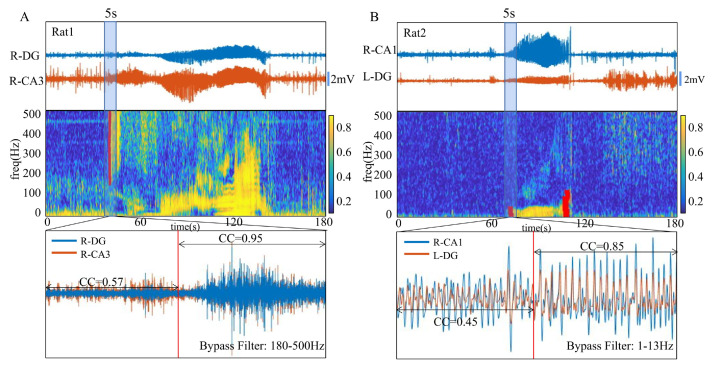
Two typical edge detection results of MSC maps. (**A**) The high-frequency band results from Rat-1. (**B**) The low-frequency band results from Rat-2. The red line is the Canny edge detection algorithm result. We subsequently enhanced the edge area, and there are obvious changes of correlation on both sides of the edge of the specific frequency band detected by the Canny edge detection algorithm.

Despite the same drug-induced seizure procedure in all rats, the results of MSC maps showed that each rat had its seizure activity mode. Two typical results are shown in Figure 3A,B. Canny edge detection showed a strong correlation change located in the high-frequency band of the MSC map of R-DG and R-CA3 channels from Rat-1 (Figure 3A). With R-CA1 and L-DG channels from Rat-2, Canny edge detection result was located in the low-frequency band (Figure 3B). We found the exact time point of the edge detection result, which in this case, appeared immediately before the seizure-like waves in the LFP signal. We further filtered the above LFP signals with the frequency band detected by the Canny edge detection algorithm to illustrate the signal correlation coefficient value between the two channels. The LFP signals from Rat-1 were filtered by frequency band of 180–500 Hz, and signals from Rat-2 were filtered by frequency band of 1–13 Hz. Both rats illustrated a marked change in CC values. By taking the detected edge as the dividing line, the CC value of Rat-1 changed from 0.57 before the edge was detected, to 0.95 after it was detected; Rat-2 changed from 0.45 to 0.85, respectively.

For a single rat, not all channels showed an increased synchronization. We defined the quantified cross-correlation strength (QCCS) as the sum of all pixel values within 5 s before and after the edge detected in the MSC map:

(13) $$QCCS=\sum_{i=p_{start}}^{p_{start}+5\;s}\sum_{f=0\;\mathrm{Hz}}^{500\;\mathrm{Hz}}V_{\mathrm{pixel}\;}\left(t,f\right)$$

where *t* and *f* represent the two cumulative dimensions of time and frequency in the MSC map, the time dimension starts from point $p_{start}$, 2.5 s before the edge, to 2.5 s after the edge. The frequency dimension ranges from 0 Hz to 500 Hz. The calculation result of QCCS is the cumulative sum of all pixel values $V_{\mathrm{pixel}\;}$ in the region.

**Figure 4:**
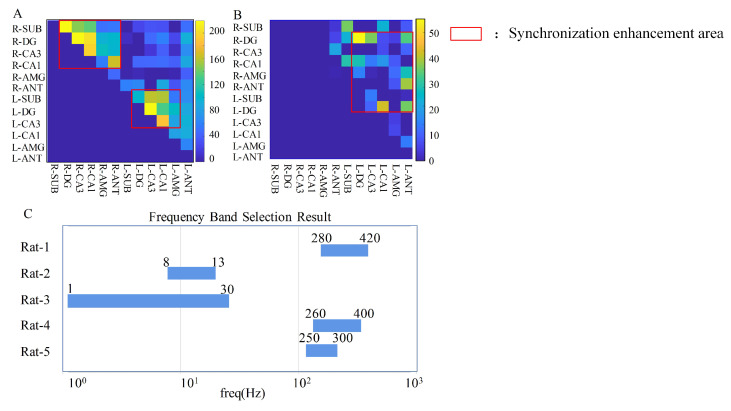
Channel and frequency band selection. (**A**) The QCCS matrix at the seizure onset of Rat-1. (**B**) The QCCS matrix at the seizure onset of Rat-2. The red box indicates the area with enhanced synchronization. (**C**) The frequency band selection results of all rats.

Figure 4A illustrates the QCCS matrix of all MSC maps during one seizure onset of Rat-1, which had a strong correlation change in the high-frequency band. The coherence enhancement was mainly in the right and left hippocampal regions of Rat-1, indicating that seizure activity may originate from the hippocampal area and be enhanced by oscillating inside the two hippocampi. Figure 4B shows the result of the seizure onset of Rat-2, which had a strong correlation change at low frequencies. The coherence enhancement of Rat-2 mainly appeared between the right and left hippocampus and with the left ANT. Therefore, the seizure activity on Rat-2 may spread wider from one hippocampus to the other and other brain areas.

After the channel selection, we further determined the frequency band by intersecting the edge results of the selected channels. We calculated the frequency band every six days to ensure stability. The final frequency band selection is shown in Figure 4C, and the details are provided in Table 2. From the results of frequency band selection, rats 1, 4, and 5 had a strong correlation change in the high-frequency band and Rat-2 and Rat-3 in the low-frequency range.

**Figure 5:**
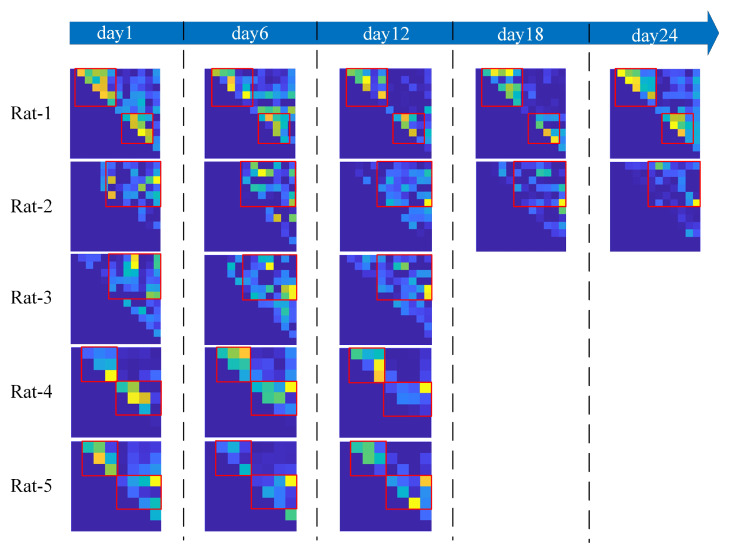
QCCS matrix changes of all rats every 6 days. The red box indicates the area with enhanced synchronization.

For the selected channels, the stability is also evaluated every six days. The QCCS matrix is used for the evaluation, and the results are shown in Figure 5. As time changes, the synchronization between some channels changes. However, the intensity of synchronization and the distribution of related sites are relatively fixed. The channel pairs with high synchronization intensity of Rat-1, Rat-4 and Rat-5 are mainly distributed in the left and right hippocampus, and the channel pairs with high synchronization intensity of Rat-2 and Rat-3 are mainly distributed between the left and right hippocampus, as shown in the red box in Figure 5. The channels are selected from the red box to ensure the stability of long-time detection while ensuring the high discrimination ability in the detection process.

### 3.3. Detection Result and Validity

**Figure 6:**
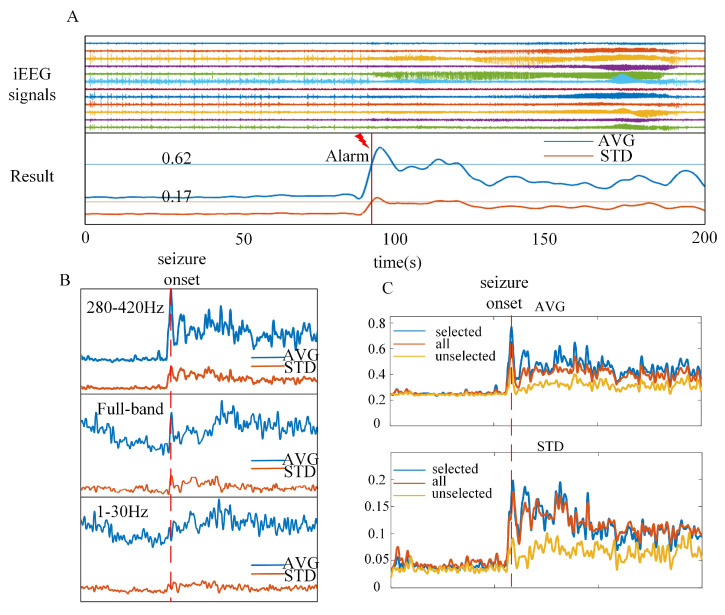
Examples of detection result and validity. (**A**) A typical example of detection results from Rat-1, the seizure onset can be detected by the thresholds. (**B**) The comparison of Rat-1 detection results in different frequency bands. (**C**) The comparison of Rat-1 detection results in different used channels.

A typical example of the detection results from Rat-1 is shown in Figure 6A. The start of seizure activity was accompanied by a sharp increase in AVG and STD. Seizure onset was identified by both the AVG and STD thresholds. Generally, the thresholds of each rat were stable after five iterations. The threshold results for all five rats are shown in Table 3. The first five seizure activities calculated the initial thresholds. The thresholds of Rat-4 are stable at initialization, and the rest of the rats have minor threshold changes.

The frequency band selection was sensitive to seizure detection. As shown in Figure 6B, we compared the AVG and STD results in three different frequency bands.The curve clearly changed at seizure onset in the high-frequency band (280–420 Hz), which is selected by MSC maps and Canny edge detection algorithm. However, the curve changes slightly at seizure onset in the full-band and low-frequency band (1–30 Hz). The frequency band selection would affect the detection accuracy, false alarm rate, delay and other results. The channel selection was also sensitive to seizure detection. As shown in Figure 6C, we compared the AVG and STD results of the selected channels, all channels and unselected channels. Using the selected channels, the curve has more significant discrimination, which is more conducive to detection.

### 3.4. Comparison Results

We used this method to detect all seizures in this study, and the results are shown in Table 4. The detection rate of all rats is 96.60%. The average detection delay time after seizure onset is 1.25 ± 0.18 s. The false alarm rate of all rats is 2.63/h. Compared with the CC feature without frequency band and channel selection, our method can greatly improve the detection effect, and the effect is the best among all comparison features. In addition, multi-channel features such as CC and MPC detection performance are better than single-channel features.

It should be noted that every feature has the possibility of optimization. This comparison only reflects this dataset’s results, which may be limited and incomprehensive.

We also compared our method with other previous works, as is shown in Table 5. The comparison methods contain the features we use for comparison. It can be seen that the increase in the number of features used can lead to an increase in detection efficiency. In-sample parameter optimization can also improve detection results. Our method uses only one single feature, but by optimizing the selection of feature, we can also achieve the desired detection results.

## 4. Discussion

Our study developed a method to find a suitable feature for seizure detection in TLE model rats. We used the MSC map and Canny edge detection to verify the synchronization change phenomenon in the seizure rats and find the strongest change frequency band. We used the maximal cross-correlation coefficient to indicate the synchronization change in a computationally efficient and appropriate manner. We extracted two features of the correlation coefficient curves and iterated the thresholds to obtain the available feature thresholds. The feature extraction method for seizure detection proposed in this paper has the following advantages: high detection rate, low detection delay, low false alarm rate. In the preprocessing step, the feature extraction method has individually adaptive but has a certain computational complexity. Therefore, in the detection step, the feature extraction results can be transformed into filtering and cross correlation calculation to reduce the computational complexity.

Because it is difficult to build a Pilocarpine-induced chronic TLE rat model, we have limited data in this study. However, we can still get some interesting phenomena. One of them is the selection of the specific frequency band and channels. Although many studies have found synchronization changes in different frequency bands [21], some studies have investigated synchronization changes by constructing networks [46,47]. However, the reason for the synchronization changes still not fully understood. In this work, we found that the epileptic transmission structure may cause the frequency band difference; for the low-frequency band between the left and right hemispheres, the high-frequency band is shown in the hemispheres. This phenomenon needs to be confirmed by more experimental samples, and more experiments and methods are needed to explain the biological mechanism. This phenomenon can help differentiate epileptic types.

Furthermore, detecting lesion sites and key nodes is also an important task in epilepsy research, especially in clinical treatment, where doctors use CT, MRI, PET [48], and other medical imaging methods to locate the suspected lesions. Electrophysiological signals are the most direct and final means of confirmation for some patients with occult lesions. Localizing epileptic lesions is difficult and depends on the location of the implanted electrodes. The graph theory [49] and causal analysis [50] methods are widely used in network analysis and attempt to analyze epileptogenic areas or key sites with a limited number of electrodes. However, the computation becomes more complex with an increase in the number of electrodes. Our method may be helpful to preliminarily determine the correlation between channels and rank the importance of channel pairs. It provides a reference for researchers, and they can select nodes of interest for analysis according to their judgment and reduce computational complexity.

Based on the limitations of the current experiment, future research into the following is warranted:

(1) Experimental verification of different species of organisms. The epilepsy characteristics of different species have similarities and differences. The synchronization of epilepsy in different individuals requires further investigation. Ultimately, the seizure detection method must to serve as an early warning and treatment for epilepsy. Our future studies will utilize the MSC map method to detect seizure activities in the epileptic brain of humans, confirming whether the method is effective.

(2) Identification of the seizure sites. Our study focused on finding the onset of seizure activity, and some important channel pairs can be selected. However, our study also had a limitation. The ten important channel pairs are changed during the progression of epilepsy, but we only used the initially selected channel pairs to detect the seizure to reduce computational complexity. The results of this detection are not superior. The location of lesions has also not been studied in detail. We can determine when the seizure begins by which sites have a strong correlation, which may be related to the location of the lesion, but further study is warranted.

(3) Dynamic analysis process. The MSC map can show the synchronization changes over the entire period, including the preictal, ictal, postictal, and interictal. It can help us to analyze the dynamic changes during seizures. Especially in recent years, the emergence of technicals such as sub-scalp EEG has made it possible to monitor patients’ seizures for a very long time [51]. The method is suitable for long-term seizure recording and can help us determine what happens in the epileptic brain.

## 5. Conclusions

This study developed a feature extraction method for seizure detection based on multi-site synchronous changes in TLE. We investigated five chronic TLE rats with 8- and 12-channel detection sites in the hippocampus and limbic system. We used the MSC map and Canny edge detection algorithm to determine the synchronization changes in the specific frequency band and channel. We developed this phenomenon into a detection method and tested the seizure data of five rats. The method achieved high performance, with an average 96.60% detection rate, 2.63/h false alarm rate, and 1.25 s detection delay. The experimental results show that synchronization is an appropriate feature for seizure detection. The MSC map and edge detection algorithm can assist in selecting a specific frequency band to enhance the detection result.

## Figures and Tables

**Table 1:** Rats and number of seizure activities.

	Seizure Activities	Monitoring Time (h)
Rat-1	67	576
Rat-2	90	672
Rat-3	32	360
Rat-4	5	336
Rat-5	12	336
Total	206	2280

**Table 2:** The frequency changes.

	Day 1	Day 6	Day 12	Day 18	Day 24
Rat-1	**280–420**	280–453	277–420	264–480	150–450
Rat-2	6–34	**8–13**	8–16	8–22	8–13
Rat-3	**1–30**	1–30	1–30	——	——
Rat-4	**260–400**	260–400	234–400	——	——
Rat-5	**250–300**	250–304	249–302	——	——

**Table 3:** Threshold iteration.

		Intial	Day 3	Day 6	Day 9	Day 12	Day 15	Day 18	Day 21	Day 24	Day 27
Rat-1	AVG	0.66	**0.62**	0.62	0.62	0.62	0.62	0.62	0.62	0.62	——
	STD	0.17	**0.17**	0.17	0.17	0.17	0.17	0.17	0.17	0.17	——
Rat-2	AVG	0.76	0.76	**0.75**	0.75	0.75	0.75	0.75	0.75	0.75	0.75
	STD	0.17	0.17	**0.17**	0.17	0.17	0.17	0.17	0.17	0.17	0.17
Rat-3	AVG	0.77	**0.71**	0.71	0.71	0.71	0.71	——	——	——	——
	STD	0.29	**0.28**	0.28	0.28	0.28	0.28	——	——	——	——
Rat-4	AVG	**0.29**	0.29	0.29	0.29	0.29	——	——	——	——	——
	STD	**0.18**	0.18	0.18	0.18	0.18	——	——	——	——	——
Rat-5	AVG	0.44	**0.44**	0.44	0.44	0.44	——	——	——	——	——
	STD	0.22	**0.28**	0.28	0.28	0.28	——	——	——	——	——

**Table 4:** Detection result.

	Detection Rate (%)	Detection Delay (s)	False Alarm Rate (1/h)
Our method	**96.60**	**1.25 ± 0.18**	**2.63**
CC	94.67	10.09 ± 9.41	5.36
MPC	91.75	9.32 ± 9.31	3.01
TP	86.41	12.49 ± 8.47	3.78
LL	85.44	9.88 ± 7.83	4.40
VAR	84.47	10.44 ± 6.72	4.69
AMP	82.52	10.95 ± 7.07	3.74
ApEn	77.18	13.24 ± 8.61	3.76
PSR	74.27	12.21 ± 7.13	2.76
MSP	73.30	25.40 ± 9.40	5.03

**Table 5:** Comparison with previous works.

	Signal Type	Used Features	In-Sample Parameter Optimization	Test Seizures	Detection Rate (%)
our method	iEEG	maximal cross correlation	YES	206	96.6
Fumeaux et al. [42]	ECoG/LFP	20 features (including univariate and multivariate, linear and nonlinear, time, and frequency domains.)	NO	202	0.962 (AUROC)
Noertjahjani et al. [43]	scalp EEG	9 features (mean, variance, standard deviation, skewness, kurtosis, minimum, maximal, correlation, energy)	YES	16	91 (Accuracy)
Schelter et al. [44]	iEEG	phase synchronization	NO	20	70 (Sensitivity)
Alaei et al. [45]	scalp EEG	mean-phase coherence	YES	>88	100 (Sensitivity)

**Figure A1:**
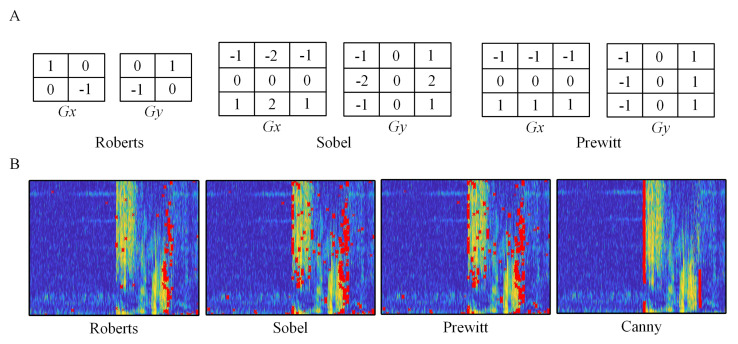
Comparison of detection effects of different edge detection algorithms. (**A**) The matrix representation of 3 algorithms: Roberts algorithm, Sobel algorithm, and Prewitt algorithm. (**B**) Comparison of edge detection effects of 4 algorithms.

## Data Availability

The data presented in this study are available in the article.

## Appendix A. Comparison of Detection Effects of Different Edge Detection Algorithms

In our study, we compared four different edge detection algorithms, including Canny, Roberts, Sobel, and Prewitt. The Canny edge detection algorithm was finally selected to compare detection effects comprehensively. The matrix representation of the other three algorithms is shown in Figure A1A. The edge detection effect is shown in Figure A1B.

## Appendix B. The Calculation Formula of Comparison Features

In our study, seven single-channel features were added for comparison. The calculation formula for these features is as follows:

Amplitude:

(A1) $$AMP=\frac{1}{N}\sum_{i=1}^{N}\left|x\left(i\right)\right|$$

Line length:

(A2) $$LL=\sum_{i=1}^{N-1}\left|x\left(i+1\right)-x\left(i\right)\right|$$

Variance:

(A3) $$VAR=\frac{1}{N}\sum_{i=1}^{N}{\left(x\left(i\right)-\bar{X}\right)}^{2}$$

Maximum slope:

(A4) $$MSP=\frac{\max(x(i))-\min(x(j))}{\left|i-j\right|}$$

Total power:

(A5) $$TP=\sum_{i=-N}^{N}{\left|x\left(i\right)\right|}^{2}$$

Power spectral ratio:

(A6) $$PSR=\frac{\mathrm{BandPower}(1-30)}{\mathrm{BandPower}(30-500)}$$

Approximate entropy:

(A7) $$ApEn=\phi_{m}-\phi_{m+1}$$

In the above formulas, x(i) represents a single channel signal, $\bar{X}$ represents the average of x(i). In the ApEn formula, the calculation formula of $\phi_{m}$ is as follows:

(A8) $$\phi_{m}={\left(N-m+1\right)}^{-1}\sum_{i=1}^{N-m+1}\log\left(C_{i}^{m}\left(r\right)\right)$$

For the reconstructed m-dimensional vector X(1), X(2),... X(m), where X(i)=[x(i),x(i+1),...x(i+m−1)], $C_{i}^{m}\left(r\right)$ counts the number of vectors satisfying $||X\left(i\right),X\left(j\right)\left|\right|\leq r\;\left(1\leq i\leq N-m+1\right)$. Generally, the value of parameter *m* is set as 2, and the value of parameter *r* is set as 0.2 times the standard deviation of data.
